# Lipid profile reversely predicts the development of dementia in patients with chronic kidney disease‐ from TMUCRD database

**DOI:** 10.1002/alz70860_102049

**Published:** 2025-12-23

**Authors:** Yi‐Chou Hou

**Affiliations:** ^1^ Cardinal Tien Hospital, New Taipei City, Taiwan; Cardinal Tien Hospital, New Taipei City, New Taipei City, Taiwan

## Abstract

**Background:**

Chronic kidney disease (CKD) is a significant global health issue often associated with dementia. Understanding the interplay between biochemical markers, comorbidities, and dementia risk in CKD patients is critical for better prevention and management.

**Methods:**

This study analyzed CKD patients from Shuang Ho Hospital and Taipei Medical University Hospital (TMUH), dividing them into dementia and non‐dementia groups. Demographic, comorbidity, and biochemical data, including hemoglobin (Hb), glomerular filtration rate (GFR), albumin, calcium, phosphorus, cholesterol, and triglycerides, were compared. Risk ratios (RR) were calculated using established clinical thresholds, and protective factors were identified based on lipid‐related parameters.

**Results:**

Patients with dementia were older and had higher rates of cardiovascular disease, heart failure, stroke, and diabetes. Significant predictors of dementia included Hb < 10 g/dL (RR: 1.608), eGFR < 30 mL/min (RR: 1.288), albumin < 3.5 g/dL (RR: 1.606), glucose > 200 mg/dL (RR: 1.301), calcium < 8.5 mg/dL (RR: 1.403), and BUN > 100 U/L (RR: 1.464). Surprisingly, elevated LDL (>100 mg/dL), total cholesterol (≥200 mg/dL), and triglycerides (≥150 mg/dL) were protective factors, with fewer dementia cases observed in patients with more protective factors.

**Conclusion:**

The study highlights the complex relationship between CKD, biochemical markers, and dementia risk. While several abnormalities increased dementia risk, elevated lipid parameters appeared protective. These findings underscore the need for tailored interventions targeting metabolic and biochemical abnormalities to reduce dementia risk in CKD patients while exploring protective mechanisms.

